# Is the endocrine research pipeline broken? A systematic evaluation of the Endocrine Society clinical practice guidelines and trial registration

**DOI:** 10.1186/s12916-015-0435-z

**Published:** 2015-08-12

**Authors:** Naykky Singh Ospina, Rene Rodriguez-Gutierrez, Juan P. Brito, William F. Young, Victor M. Montori

**Affiliations:** Knowledge and Evaluation Research Unit, Division of Endocrinology, Diabetes, Metabolism and Nutrition, Mayo Clinic, 200 1st Street SW, Rochester, MN 55905 USA; Division of Endocrinology, Diabetes, Metabolism and Nutrition, Mayo Clinic, Rochester, MN USA

**Keywords:** Endocrinology guidelines, Endocrine Society, GRADE system, Very low quality evidence

## Abstract

**Background:**

Very low quality (VLQ) evidence translates into very low confidence in the balance of risk and benefits based on the estimates drawn from the body of evidence. Consequently, this assessment highlights gaps in the research evidence, i.e. knowledge gaps, for important clinical questions. In this way, expert guideline panels identify priority knowledge gaps that, arguably, should inform the research agenda and prioritize scarce research economical resources. The extent to which the research agenda reflects the knowledge gaps identified in clinical practice guidelines is unknown.

**Methods:**

A systematic evaluation of the Endocrine Society (ES) clinical practice guidelines portfolio from 2008 to 2014 was conducted with the objectives to identify (1) recommendations in the ES clinical practice guidelines based on VLQ evidence reflecting knowledge gaps in endocrinology, and (2) active research designed to address these gaps by searching the clinical trial registry, clinicaltrials.gov, using terms describing patients (diseases), interventions, comparison, and outcomes.

**Results:**

In 25 ES guidelines, we found 660 recommendations, of which 131 (20 %) were supported by VLQ evidence. Clinical trialists are attempting to answer 28 (21 %) of these knowledge gaps by performing 69 clinical trials.

**Conclusion:**

The research enterprise is addressing one in five knowledge gaps identified in clinical practice recommendations in endocrinology. These findings suggest an inefficiency in the allocation of very scarce research economical resources. Linking the research agenda to evidence gaps in clinical practice guidelines may improve both the efficiency of the research enterprise and the translation of evidence into more confident clinical practice.

## Background

The Endocrine Society (ES) has created clinical practice guidelines to aid clinicians in the care of patients with endocrine disorders. In 2005, the ES adopted the Grading of Recommendations, Assessment, Development and Evaluation (GRADE) Group system [1–4]. The GRADE approach rates the panel’s confidence in the risk estimates of favorable and unfavorable outcomes. This confidence in the estimates is captured by classifying the quality of the body of evidence supporting a recommendation into one of four categories: high, moderate, low, and very low quality (VLQ) [1–4]. VLQ evidence results when the body of evidence is comprised of studies at high risk of bias, yielding imprecise results or results of indirect relevance to the recommendation, and/or results that are inconsistent across studies or are not fully reported. VLQ evidence translates into very low confidence in the balance of risk and benefits based on the estimates drawn from the body of evidence [3, 4]. Consequently, this assessment highlights gaps in the research evidence, i.e. knowledge gaps, for important clinical questions. Since these knowledge gaps affect the assessments about the balance of benefits and harms of alternative courses of action they reduce our confidence that patients will be better off were they to receive care consistent with recommendations based on VLQ evidence.

A recent study showed that low to very low quality evidence, largely derived from small observational studies, supported most endocrinology guideline recommendations [5, 6]. To this extent, guidelines that explicitly account for confidence in estimates from the body of evidence, such as ES guidelines, can be used to identify important knowledge gaps and guide the research agenda. This is particularly important in the face of scarce, research dollars. Moreover, it has been previously estimated that 85 % of research is of low impact or wasted mainly due to being unnecessary, poorly designed, biased, unusable, incompletely published, or simply addressing the wrong research question. A better connection between knowledge gaps identified in practice guidelines and the research enterprise could reduce research waste [7–12]. To explore the integrity of the pipeline connecting knowledge gaps with ongoing research, we conducted a study using ES guidelines.

## Methods

We conducted a systematic evaluation of the available ES clinical practice guidelines to identify clinical recommendations that are based on VLQ evidence and that potentially reflect knowledge gaps in endocrinology. Using the ES guideline web site, we identified and retrieved all ES clinical practice guidelines issued from 2008 to 2014 [13–38]. For each guideline, two reviewers working independently searched and extracted the number of graded recommendations in each guideline and those rated as based on VLQ evidence. Guideline panels following the GRADE approach, as is the case with ES guidelines, rate evidence as VLQ when the body of evidence produces estimates about which we have very low confidence. Studies produce low-confidence estimates when they are at high risk of bias, produce results that are of indirect relevance to the recommendation, imprecise or inconsistent, or when there is evidence of incomplete and biased reporting [3, 4, 39].

Because of problems with classification, some recommendations based on VLQ evidence should not have been graded as they represent best practice statements in which there is no sensible alternative. In this sense, best practice recommendations are thought to do substantially more good than harm (or vice versa) and therefore no one would consider doing a study to definitively establish the answer to the implicit question [40]. Examples of best practice recommendations include: (1) “We suggest that female-to-male transsexual persons evaluate the risks and benefits of including total hysterectomy and oophorectomy as part of sex reassignment surgery”, and (2) “In patients presenting with heart failure, initial assessment should be made of the patient’s ability to perform routine/desired activities of daily living” [20, 38, 41]. After excluding these, the crude inter-observer agreement for the identification of recommendations based of VLQ evidence was 96 %.

We then identified the research questions relevant to these knowledge gaps in terms of patients, interventions, comparisons, and outcomes (PICO). For each VLQ evidence item, we drafted a research question that a clinical trial could answer using the PICO format (e.g. Patients – patients with large adrenal pheochromocytomas; Intervention – minimally invasive adrenalectomy; Comparison – open resection adrenalectomy; Outcome – complete tumor resection, tumor rupture avoidance, and local recurrence rates) [1, 35, 39]. The objective of this step was to make the underlying questions explicit thus ensuring reproducibility of our methods. To calibrate this process, two researchers independently produced these questions for 11 recommendations with an initial agreement of 80 %. This process was repeated until the agreement was 100 %, achieved after 20 recommendations.

We then searched the clinicaltrials.gov database for active studies (randomized and observational) addressing the questions identified from the guidelines. Since 2000, this registry has emerged as the most complete including trials from 188 countries. A clinical trial was deemed eligible if it addressed the PICO question with at least one of the necessary outcomes. For each eligible clinical trial, we extracted the study design, source of funding, year of entry, and the sample size. Although we did not look for publication of results, we excluded trials completed 5 or more years prior to the date of guideline publication. Reviewers working independently searched the clinical trial registry until 100 % agreement, a point reached after searching for 20 questions. A single reviewer completed the search for the rest of the questions.

### Statistical analysis

We performed a descriptive analysis and summarized continuous variables as mean (SD) and presented percentages in cases of categorical variables.

## Results

**Fig. 1:**
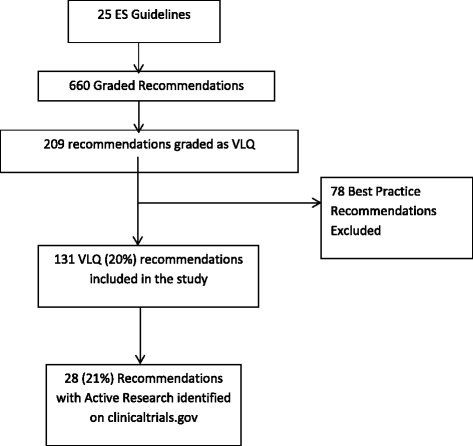
Selection process. ES, Endocrine Society; VLQ, Very low quality

**Fig. 2:**
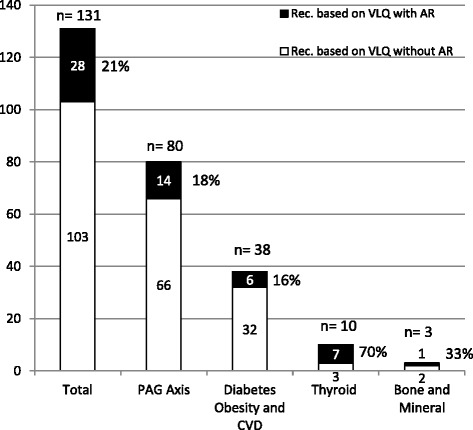
Proportion of recommendations based on VLQ evidence with and without active research studies. VLQ, Very low quality; Rec., Recommendation; AR., Active research; PAG, Pituitary adrenal and gonad axis; CVD, Cardiovascular disease. Numbers in percentage correspond to recommendations based on VLQ evidence with active research

We identified 25 Clinical Practice Guidelines from the ES with 660 recommendations, of which 209 (32 %) were supported by VLQ evidence [8–32]. After excluding 78 (12 %) best practice statements, the total was 131 (20 %) recommendations based on VLQ evidence (Fig. 1). The majority of the guidelines supported the care of patients with pituitary, gonadal, and adrenal disorders [13], and most recommendations supported by VLQ evidence came from these guidelines (24 %; Fig. 2).

Active research was identified for 28 (21 %) of these 131 recommendations represented by 69 clinical studies (mean of 2.5 studies per each recommendation – when taking into consideration those with active research; Fig. 1). Of these, 35 (51 %) were randomized trials and 34 (49 %) were observational. Thirteen reported industry funding and six were multicenter studies. Of the 69 active studies, 42 (60 %) were in the guidelines of thyroid dysfunction during pregnancy, testosterone therapy in adult men, and diagnosis of Cushing syndrome which had a total of 32 recommendations supported by VLQ evidence, of which 15 had at least one active research study.

Most of the identified clinical trials addressed knowledge gaps affecting recommendations for patients with thyroid disorders (70 %); the least dealt with were gaps in the evidence for care of patients with diabetes, obesity, and cardiovascular disease (16 %; Fig. 2).

## Discussion

Important knowledge gaps are evident in 10 of every 50 ES clinical practice guideline recommendations, of which the research enterprise is actively addressing, at best, two. Moreover, of the active trials, 60 % are trying to improve the quality of evidence in only three of the 25 guidelines. In some cases, several studies are actively addressing the same question. The research enterprise, thus characterized, poorly reflects the knowledge gaps at the frontline of endocrine practice, covering these gaps incompletely and sometimes redundantly. Multiple explanations exist for these observations. The research enterprise may not be aware of these gaps because funding agencies and researchers do not use clinical practice guidelines to identify knowledge gaps, or they may be responding to these gaps with basic studies that are not in the registry. Alternatively, ES expert ratings of confidence in estimates evidence have not been explicitly communicated to researchers and funders to facilitate the development of a practice-relevant research agenda.

This cross-sectional study contributes to the understanding of a seemingly broken research pipeline. However, there are dynamic aspects that only a longitudinal study could answer. For example, in its 2014 version, the ES guideline on Androgen Therapy in Women had seven recommendations based on VLQ evidence, all new since its 2006 version [42]. Conversely, the evidence supporting two of their 2006 recommendations was upgraded in 2014 from warranting very low to warranting low confidence in the estimates [38, 42]. How these changes result from the manner in which the research enterprise responds to knowledge gaps merits further study.

To our knowledge, there is no comprehensive repository of knowledge gaps in endocrinology. This study partially reflects the coverage of the current ES guideline portfolio based on our selection of recommendations supported by VLQ evidence, instead of the much larger set of recommendations based on low quality evidence. A key advantage of the portfolio that enabled this study is the ES’s early adoption of GRADE, which enables the separation of the rating of evidence (used here) from the grading of the strength of recommendation [4]. Further, we used the clinicaltrials.gov registry which, despite being the largest, may have missed research (e.g. well-designed observational studies, not registered studies), particularly outside the United States. Additionally, studies published after the publication of the clinical guidelines that could have assessed these gaps were not part of our analysis. Nevertheless, we identified knowledge gaps in the ES clinical practice guidelines in duplicate and performed a search of clinical trials to address them after high reproducibility was achieved between each of the independent reviewers.

We are also aware that, even though panels may be the best way to identify research gaps, it could be easily argued that this does not automatically mean that trials should be conducted in those areas, e.g. the needed studies might be prohibitively expensive, research gaps might be difficult to study, or the science field is not developed enough to support the conduct of clinical trials. On the other hand, panels choose areas of clinical relevance to formulate recommendations. Often, these represent ongoing practice, which is unlikely to be supported solely by mechanistic hypotheses or basic science data. While we do not know the extent to which our findings apply to other areas of medicine, they may very well represent a trigger to examine the research pipeline and improve the quality and relevance of the evidence. A functional pipeline connecting evidence to recommendations and back can ultimately better support decision making by patients, clinicians, policy makers, and funding agencies.

## Conclusion

Researchers are addressing only one in five knowledge gaps identified in clinical practice recommendations in endocrinology. Linking the research agenda to evidence gaps in guidelines may improve both the efficiency of the research enterprise and the translation of evidence into practice by increasing the value and reducing the waste in research.

## Abbreviations

ES: Endocrine Society
GRADE: Grading of Recommendations, Assessment, Development and Evaluation System
PICO: Patient, Intervention/Exposure, Comparison and Outcome
VLQ: Very low quality
